# Psychrophilic Enzymes: From Folding to Function and Biotechnology

**DOI:** 10.1155/2013/512840

**Published:** 2013-01-17

**Authors:** Georges Feller

**Affiliations:** Laboratory of Biochemistry, Centre for Protein Engineering, Institute of Chemistry, University of Liège, B6a, 4000 Liège, Belgium

## Abstract

Psychrophiles thriving permanently at near-zero temperatures synthesize cold-active enzymes to sustain their cell cycle. Genome sequences, proteomic, and transcriptomic studies suggest various adaptive features to maintain adequate translation and proper protein folding under cold conditions. Most psychrophilic enzymes optimize a high activity at low temperature at the expense of substrate affinity, therefore reducing the free energy barrier of the transition state. Furthermore, a weak temperature dependence of activity ensures moderate reduction of the catalytic activity in the cold. In these naturally evolved enzymes, the optimization to low temperature activity is reached via destabilization of the structures bearing the active site or by destabilization of the whole molecule. This involves a reduction in the number and strength of all types of weak interactions or the disappearance of stability factors, resulting in improved dynamics of active site residues in the cold. These enzymes are already used in many biotechnological applications requiring high activity at mild temperatures or fast heat-inactivation rate. Several open questions in the field are also highlighted.

## 1. Introduction

**Figure 1:**
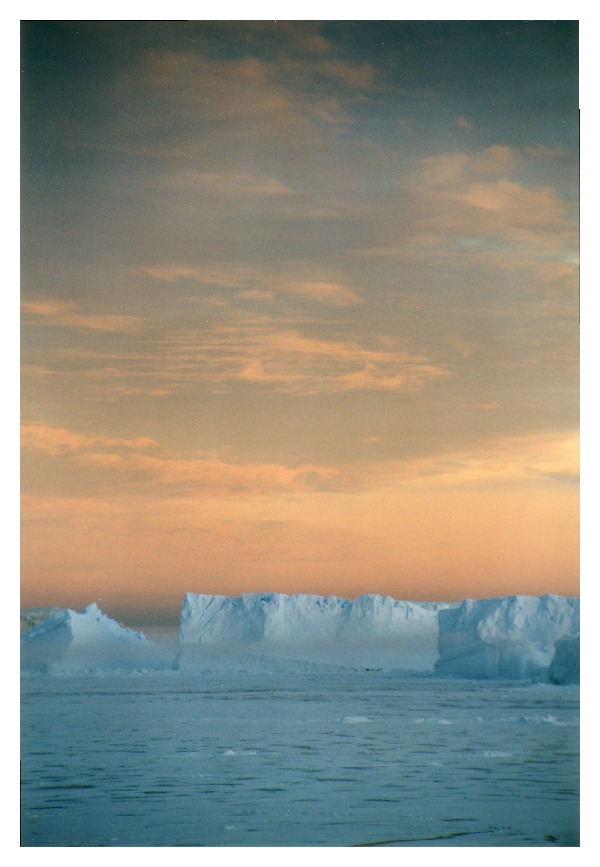
Harsh conditions and beautiful landscape. Extremely cold environments are not sterile as exemplified by polar fish thriving below the icepack or by active microorganisms found between sea ice crystals (courtesy of Dr. J. C. Marx).

“Coping with our cold planet” [1], the title of this recent review unambiguously stresses a frequently overlooked aspect: the Earth's biosphere is predominantly cold and permanently exposed to temperatures below 5°C. Such low mean temperature mainly arises from the fact that 71% of the Earth's surface is covered by oceans that have a constant temperature of 2–4°C below 1000 m depth, irrespective of the latitude. The polar regions account for another 15%, to which the glacier and alpine regions must be added as well as the permafrost representing more than 20% of terrestrial soils. Although inhospitable, all these low temperature biotopes have been successfully colonized by cold-adapted organisms (Figure 1). Psychrophiles thrive in permanently cold environments (in thermal equilibrium with the medium) and even at subzero temperatures in supercooled liquid water. Such extremely cold conditions are encountered, for instance, in salty cryopegs at −10°C in the Arctic permafrost [2, 3], in the brine veins between polar sea ice crystals at −20°C [4–6], or in supercooled cloud droplets [7, 8]. Unusual microbiotopes have also been described, such as porous rocks in Antarctic dry valleys hosting microbial communities surviving at −60°C [9, 10]. Cryoconite holes on glacier surfaces represent another permanently cold biotope hosting complex microbial communities [11, 12]. These examples highlight the unsuspected ability of psychrophiles to adapt to low temperatures.

Psychrophiles include a large range of representatives from all three domains: *Bacteria*, *Archaea*, and *Eukarya*. Psychrophiles are mainly represented by microorganisms such as bacteria [13–16], archaea [17], algae [18], or yeast [19] but also by plants and animals [20, 21]. Notably, polar fish thriving beneath the icepack are the biggest psychrophiles [22–24]. Glacier ice worms are also worth mentioning as they complete their life cycle exclusively in glacier ice [25]. All these examples illustrate that psychrophiles are the most abundant extremophiles in terms of biomass, diversity, and distribution. 

Psychrophiles do not merely survive or endure such extremely inhospitable conditions but are irreversibly adapted to these environments, as most of them are unable to grow at mild (or mesophilic) temperatures. These extremophiles represent much more than a biological curiosity. Psychrophiles and their biomolecules have already found various applications but their role in biogeochemical cycles is frequently underestimated: the huge biotope volume of oceans represents the largest reservoir of psychrophiles contributing to matter cycling. Various aspects of their ecology, physiology, and molecular adaptations have been reported in previous publications [26–30].

Life in cold environments requires a vast array of adaptive features at nearly all levels of the cell architecture and function. Indeed, cold exerts severe physicochemical constraints on living organisms including increased water viscosity, decreased molecular diffusion rates, reduced biochemical reaction rates, perturbation of weak interactions driving molecular recognition and interaction, strengthening of hydrogen bonds that, for instance, stabilize inhibitory nucleic acid structures, increased solubility of gases, and stability of toxic metabolites as well as reduced fluidity of cellular membranes [1, 31, 32]. Adaptive features to these constraints are frequently detected in the genome sequence of psychrophilic microorganisms [33–36]. Indeed, several genomes from psychrophilic bacteria and archea have been sequenced [37–46] and recently also the first genome of a polar eukaryotic microalga has been reported [47]. In this respect, recent improvements in high throughput sequencing have produced large amounts of data but most of these genomes remain to be analyzed [36, 48–55].

However, a key determinant of adaptation to life in the cold lies in the protein function that drives microbial metabolism and cell cycle. Pioneering studies of psychrophiles at the molecular level were mainly focused on cold-active enzymes because this aspect was regarded as a prerequisite to the environmental adaptation. It has been shown that the high level of specific activity at low temperature of cold-adapted enzymes is a key adaptation to compensate for the exponential decrease in chemical reaction rates as the temperature is reduced. Such high biocatalytic activity arises from the disappearance of various noncovalent stabilizing interactions, resulting in an improved flexibility of the enzyme conformation. It should be noted that this adaptive feature is genetically encoded within the protein sequence and results from a long-term selection. As a general picture, psychrophilic enzymes are all faced to a main constraint, to be active at low temperatures, but the ways to reach this goal are quite diverse. The main functional and structural adaptive properties of cold-active enzymes are presented here as well as the recent advances related to their synthesis, folding and biotechnological applications. This paper is based on updates of previous publications [56–64].

## 2. Protein Synthesis and Folding

For many years, protein synthesis and protein folding have been regarded as temperature-sensitive cellular processes that severely restrict microbial growth at low temperature in the absence of specific adaptations [65–70]. Despite this well-recognized limitation, the challenge of protein synthesis and folding in psychrophiles has been addressed only recently, mainly via proteomics and, to a lesser extent, transcriptomics [36, 62]. These approaches have produced a huge amount of data with, however, contrasted patterns of cold adaptation [43, 55, 71–95]. Indeed, proteins differentially expressed at low temperature, either cold induced or cold repressed, do not constitute a conserved set of proteins in terms of identification and expression levels in these organisms. It has been suggested that cold adaptation superimposes on preexisting cellular organization and, accordingly, that the adaptive strategies may differ between the various psychrophilic organisms [35, 62]. Nevertheless, and as far as protein synthesis and folding are concerned, some general trends have been noted.

### 2.1. Translation

In the case of the Antarctic bacterium *P. haloplanktis*, 30% of the upregulated proteins at 4°C were found to be directly related to protein synthesis [83]. It was concluded that protein synthesis may be a rate-limiting step for growth in the cold, therefore inducing a compensatory cellular response. A similar pattern is indeed observed in several cold-adapted bacteria [76, 78, 80, 84, 85], which overexpress proteic components involved in translation. More specifically, the synthesis of ribosomal proteins and of RNA chaperones [96, 97] appears to be stimulated at low temperature [43, 55, 75, 78–81, 84]. In the archaeon *M. burtonii*, a notable feature is the upregulation of genes involved in maintaining RNA in a state suitable for translation and for enabling translation initiation [86]. At the genome level, an interesting observation is the relatively high number of rRNA genes and of tRNA genes (up to 106 genes, sometimes organized in long runs of repeated sequences), at least in *P. haloplanktis* [39], *C. psychrerythraea* [40], and *P. ingrahamii* [42]. This could reflect the need for a high capacity for translation in the cold. In addition, RNA helicases have been found to be overexpressed at low temperature in many psychrophilic microorganisms such as *M. burtonii* [98], *E. sibiricum* [43], *S. alaskensis* [84], *P. arcticus* [80, 81], *S. denitrificans* [55], and *P. haloplanktis* [83]. These helicases may help to unwind the RNA secondary structures for efficient translation in the cold [99]. These examples indicate that psychrophiles have indeed evolved adaptive mechanisms to optimize protein synthesis at low temperature.

### 2.2. Folding Assistance

Some interesting reports have revealed that cold-adapted chaperones such as DnaK [100, 101] and GroEL [102–104] expressed in *E. coli* provide cold-resistance and improve growth of the mesophilic bacterium at low temperature, therefore highlighting the crucial involvement of chaperones in temperature adaptation of microorganisms.

**Figure 2:**
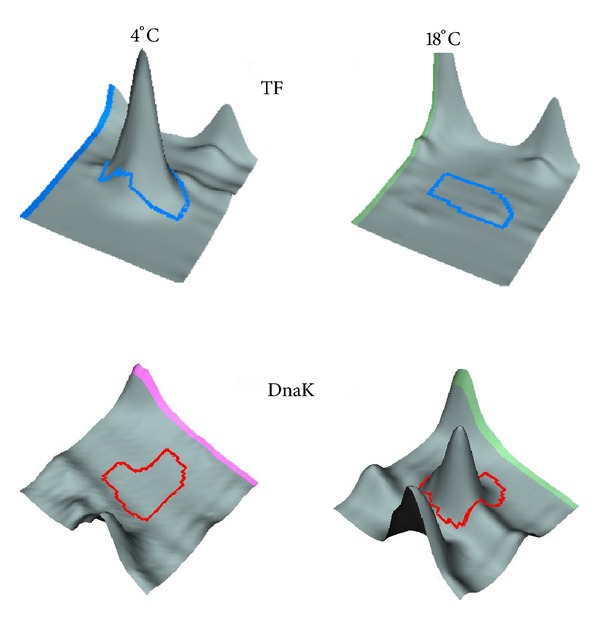
Comparative analysis of electrophoretic spots containing the trigger factor TF and DnaK from *P. haloplanktis* grown at 4°C (left panels) and 18°C (right panels) illustrating the overexpression of TF (a cold shock protein) and the downregulation of DnaK (a heat shock protein) at low temperature. Adapted from [83].

Amongst the various chaperones involved in protein folding assistance [105], the ribosome-bound trigger factor (TF) deserves a special mention. TF is the first chaperone interacting cotranslationally with virtually all nascent polypeptides synthesized by the ribosome, and most small proteins (~70% of total) may fold rapidly upon synthesis without further assistance. Interestingly, TF is a cold shock protein in *E. coli* [106], whereas the other chaperones are well-known heat shock proteins (HSPs). This significant distinction appears to be of prime importance for psychrophiles. As a matter of fact, overexpression of TF at low temperature has been observed in several cold-adapted microorganisms [75, 79, 83, 87] while downregulation of HSP chaperones has been noted [43, 73, 88]. Considering such imbalance in the chaperone machinery (Figure 2), it appears that TF rescues the chaperone function and should be regarded as the primary chaperone in these strains [83]. The downregulation of HSP chaperones in the cold suggests that these folding assistants are mainly synthesized at transiently higher environmental temperatures [62]. 

By contrast, repression of TF synthesis or upregulation of HSP chaperonins has been also reported [80, 81, 84]. Furthermore, *S. alaskensis* possesses two sets of *dnaK-dnaJ-grpE* gene clusters (DnaK and its cochaperones). Quantitative proteomics has suggested that one set functions as a low temperature chaperone system, whereas the other set functions at higher growth temperatures [84]. Obviously, distinct strategies have been adopted by psychrophiles to assist proper protein folding.

### 2.3. Proline Isomerization

**Figure 3:**
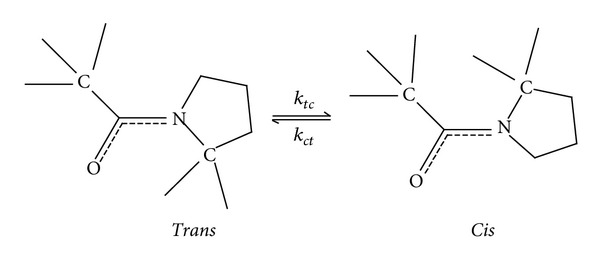
*Trans-* and *cis-* isomers of a peptidyl-prolyl peptide bond. Reprinted with permission from [110] © 2009 American Chemical Society.

The *cis-trans-*isomerization of the peptidyl-prolyl bond, that is, the peptide bond preceding a proline residue (Figure 3), is an intrinsically slow reaction. As a result, proline isomerization is a rate-limiting step for the folding of most proteins [107]. More specifically for psychrophilic proteins, the spontaneous rate of the proline isomerization reaction should also be slowed down at low temperature. Interestingly, many cold-adapted proteins tend to possess a reduced proline content [57, 60, 108] that should attenuate the negative effect of proline isomerization on folding.

In addition, living cells are equipped with specialized catalysts, prolyl isomerases (PPiases), which accelerate the isomerization process. Very significantly, PPiases are overexpressed at low temperature in the proteome of most psychrophilic bacteria analyzed so far, and sometimes at very high levels. This involves cytoplasmic PPiases from the cyclophilin family [71, 72, 80, 83], a parvulin-type PPiase [84], and FKBP-type PPiases [71, 109]. Furthermore, the above-mentioned trigger factor contains a PPiase domain and is overexpressed in several psychrophiles. Such recurrent observations strongly suggest that protein folding is a rate-limiting step for psychrophiles, which induces a cellular response aimed at facilitating and accelerating the slowest event in the acquisition of the biologically active conformation of proteins. 

### 2.4. Folding Rate

Following synthesis by the ribosome, the rate of protein folding is intuitively expected to be slowed down at low temperature. In the case of thermophilic proteins, it has been shown that their rate of folding does not differ from the folding rate of mesophilic proteins, whereas thermophilic proteins unfold very slowly therefore revealing the kinetic origin of their stability [111–113]. Such comparative analyses are currently lacking for psychrophilic proteins. Nevertheless, the folding and unfolding rate constants have been recorded for a psychrophilic alpha-amylase and for its stabilized mutants [114]. It was found that despite large differences in stability, the folding rate of these proteins is similar, whereas the unfolding rates correlate with stability. It seems therefore that the folding rate of this cold-adapted enzyme is not altered. There is an obvious need for deeper biophysical studies on protein folding at low temperature.

## 3. Psychrophilic Enzymes

The previous sections have highlighted the current issues in protein folding at low temperature. The next sections will mainly concentrate on the enzyme function in psychrophiles which occupies a central role in cold adaptation of living organisms. 

### 3.1. 3D Structures of Cold-Active Enzymes

Crystal structures of psychrophilic enzymes were of prime importance to investigate the properties of these cold-active catalysts.  Table 1 provides an overview of representative crystal structures. To date, only one report of assignments for NMR structure determination has been published for a thiol-disulfide oxidoreductase from an Antarctic bacterium [115].  Table 1 illustrates that the available structures cover many pathways of the cell metabolism while membrane proteins, for instance, are still lacking. It is worth mentioning that all these structures are closely homologous to mesophilic counterparts: cold-active enzymes only differ by discrete changes that are responsible for their specific properties.

### 3.2. The Low Temperature Challenge

The challenge for a thermophilic enzyme is easily understood: to remain stable and active at high temperatures. By contrast, the challenge for a psychrophilic enzyme is more subtle. Low temperatures strongly reduce the rate of nearly all enzyme-catalyzed reactions and, furthermore, slow down molecular motions associated with protein function. As far as activity is concerned, the catalytic constant *k*_cat_ corresponds to the maximum number of substrate molecules converted to product per active site per unit of time, and the temperature dependence of the catalytic rate constant is given by the relation

(1) $$\begin{array}{c} k_{\text{cat}}=\kappa \frac{k_{B}T}{h}e^{-\Delta G^{\#}/RT}. \end{array}$$

In this equation, *κ* is the transmission coefficient generally close to 1, *k*_*B*_ is the Bolzmann constant (1.38 × 10^−23^ J K^−1^), *h* the Planck constant (6.63 × 10^−34^ J s), *R* the universal gas constant (8.31 J K^−1^ mol^−1^), and Δ*G* ^#^ the free energy of activation or the variation of the Gibbs energy between the activated enzyme-substrate complex ES* and the ground state ES. Accordingly, the activity constant *k*_cat_ is exponentially dependent on the temperature. As a rule of thumb, for a biochemical reaction catalyzed by a mesophilic enzyme (from bacteria or warm-blooded animals), a drop in temperature from 37°C to 0°C results in a 20 to 80 times lower activity. This is the main factor preventing the growth of nonadapted organisms at low temperatures.

**Figure 4:**
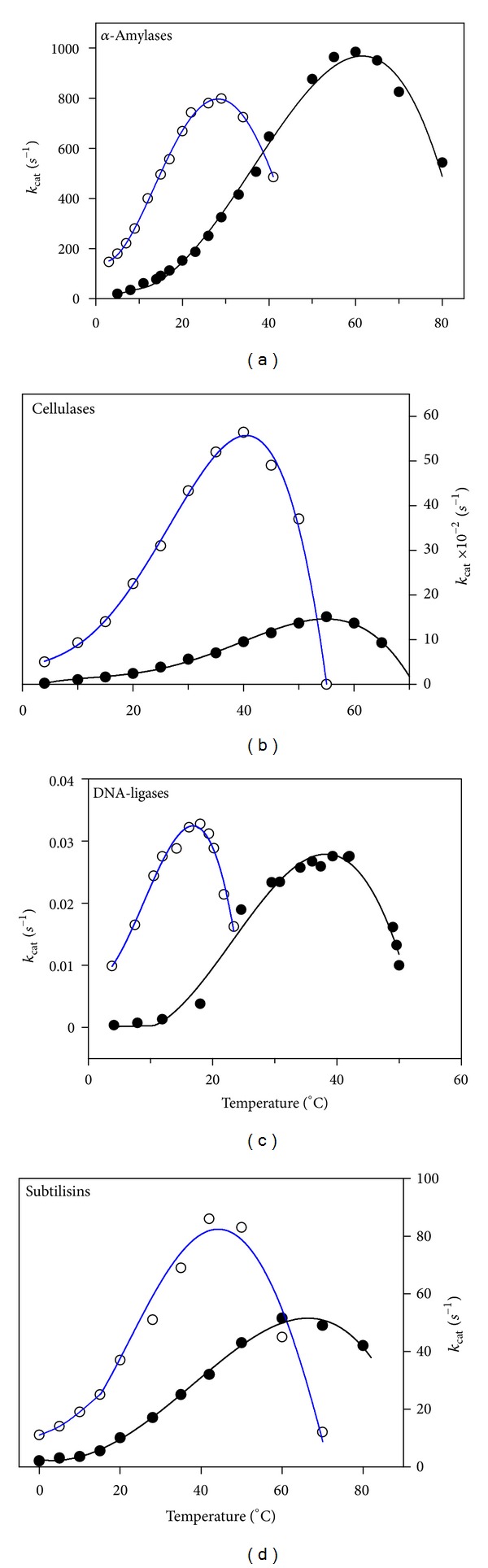
Temperature dependence of activity. The activity of psychrophilic (open symbols, blue lines) and mesophilic (closed symbols) enzymes recorded at various temperatures illustrates the main properties of cold-adapted enzymes: cold activity and heat lability. Adapted from [32, 156–158].

The effect of temperature on the activity of psychrophilic and mesophilic enzymes is illustrated in Figure 4. Equation (1) is only valid for the exponential rise of activity with temperature on the left limb of the curves. Models have been proposed to simulate the effects of heat-inactivation on activity [152–154] and to take the viscosity of the medium into account [108]. This figure reveals at least two basic features of cold adaptation. (i) In order to compensate for the slow reaction rates at low temperatures, psychrophiles synthesize enzymes having an up to tenfold higher specific activity in this temperature range. This is in fact the main physiological adaptation to cold at the enzyme level; (ii) The temperature for apparent maximal activity for cold-active enzymes is shifted towards low temperatures, reflecting the weak stability of these proteins and their unfolding and inactivation at moderate temperatures. A compilation of 155 characterized psychrophilic enzymes has been reported [155] and can be consulted (http://www2.ulg.ac.be/biochlab/publications_022.htm). 

Many observations similar to those shown in Figure 4 have suggested relationships between the activity of the enzyme, the flexibility of the protein, and its stability. Indeed, the high activity at low temperatures seems to arise from an increased flexibility of the protein structure, especially at temperatures that strongly slow down molecular motions, but the consequence of this improved mobility of the protein structure is of course a weak stability. These tradeoffs are developed in the next sections.

## 4. Cold-Adapted Activity

### 4.1. Heat-Labile and Unstable Enzymes

**Figure 5:**
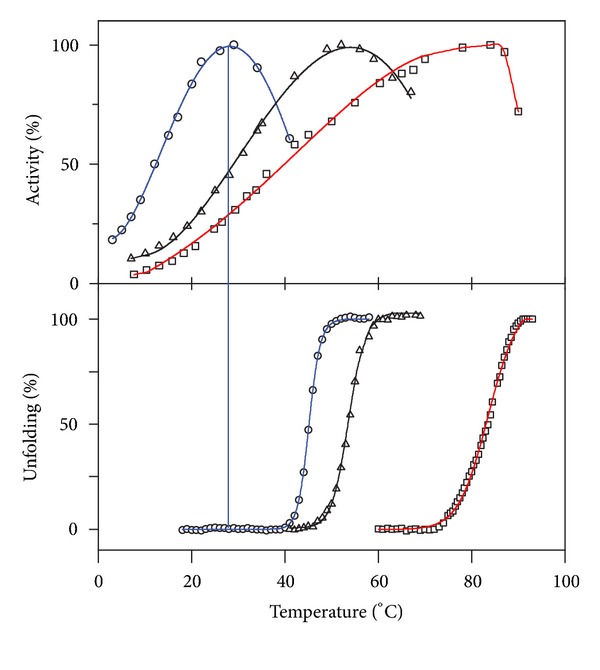
Inactivation and unfolding of psychrophilic enzymes. The activity of most psychrophilic enzymes (upper panel, blue curve) is inactivated by temperature before unfolding of the protein structure (lower panel) illustrating the pronounced heat lability of the active site. By contrast, inactivation of mesophilic (black curves) or thermophilic (red curves) enzymes closely corresponds to the beginning of protein unfolding. Adapted from [160].

Most psychrophilic enzymes share at least one property: a heat-labile activity, irrespective of the protein structural stability. Furthermore, the active site appears to be the most heat-labile structural element of these proteins [158–161].  Figure 5 illustrates this significant difference between the stability of the active site and the stability of the structure. The lower panel shows the stability of the structure as recorded by fluorescence. As expected, the structure of the cold-active enzyme is less stable than that of the mesophilic and thermophilic counterparts. In the upper panel, the activity is recorded under the same experimental conditions and it can be seen that the mesophilic and thermophilic enzymes initiate heat-inactivation when the protein starts to unfold. By contrast, activity of the cold-active enzyme is lost before the protein unfolds. This means that the active site is even more heat-labile than the whole protein structure. All these aspects point to a very unstable and flexible active site and illustrate a central concept in cold adaptation: localized increases in flexibility at the active site are responsible for the high but heat-labile activity [162], whereas other regions of the enzyme might or might not be characterized by low stability when not involved in catalysis [163]. For instance, psychrophilic carbonic anhydrase [164] and isocitrate dehydrogenase [133] are highly stable enzymes with, however, improved flexibility in regions driving catalysis. In multidomain psychrophilic enzymes containing a catalytic and a noncatalytic domain, the catalytic domain is always heat labile, whereas the noncatalytic domain can be as stable as mesophilic proteins [165–167].

### 4.2. Active Site Architecture

**Figure 6:**
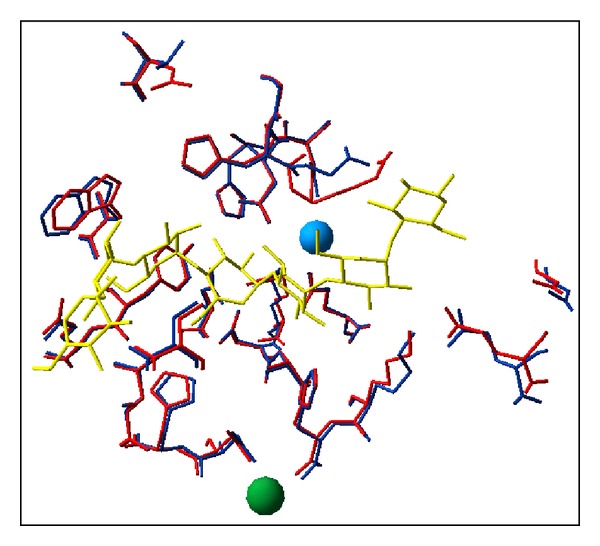
Structure of the active site. Superimposition of the active site residues in psychrophilic (blue) and mesophilic *α*-amylases (red). The chloride and calcium ions are shown as blue and green spheres, respectively. The 24 residues performing direct or water-mediated interactions with a substrate analog (yellow) are identical and superimpose almost perfectly within the resolution of the structures, demonstrating a structural identity in these psychrophilic and mesophilic enzymes [59].

With the availability of crystal structures, the cold-active catalytic centers of psychrophilic enzymes were carefully scrutinized. The first basic observation is that all side chains involved in the catalytic mechanism are strictly conserved. This aspect is not really surprising as the specific reaction mechanism of enzymes is not prone to drastic variation. Furthermore, comparison of the first X-ray structure of a psychrophilic enzyme, a cold-active *α*-amylase [116, 117], and of its closest mesophilic structural homologue, both in complex with an inhibitor mimicking the transition state intermediate [168, 169], has revealed that all 24 residues forming the catalytic cleft are strictly conserved in the cold-active *α*-amylase (Figure 6). This outstanding example of active site identity demonstrates that the specific properties of psychrophilic enzymes can be reached without any amino acid substitution in the reaction center. As a consequence, changes occurring elsewhere in the molecule are responsible for optimization of the catalytic parameters and improved dynamic of active site residues.

Nevertheless, significant structural adjustments at the active site of psychrophilic enzymes have been frequently reported. In many cases, a larger opening of the catalytic cleft is observed [142] and is achieved by various ways, including replacement of bulky side chains for smaller groups, distinct conformation of the loops bordering the active site, or small deletions in these loops, as illustrated by a cold-active citrate synthase [124]. In the case of a Ca^2+^, Zn^2+^  protease from a psychrophilic *Pseudomonas* species, an additional bound Ca^2+^ ion pulls the backbone forming the entrance of the site and markedly increases its accessibility when compared with the mesophilic homologue [122]. As a result of such a better accessibility, cold-active enzymes can accommodate substrates at lower energy cost, as far as the conformational changes are concerned, and therefore reduce the activation energy required for the formation of the enzyme-substrate complex [170]. The larger active site may also facilitate easier release and exit of products and thus may alleviate the effect of a rate-limiting step on the reaction rate [134, 171]. 

In addition, differences in electrostatic potentials in and around the active site of psychrophilic enzymes appear to be a crucial parameter for activity at low temperatures. Electrostatic surface potentials generated by charged and polar groups are an essential component of the catalytic mechanism at various stages: as the potential extends out into the medium, a substrate can be oriented and attracted before any contact between enzyme and substrate occurs. Interestingly, the cold-active citrate synthase [124], malate dehydrogenase [131], uracil-DNA glycosylase [151, 172, 173], elastase [174], and trypsin [175–177] are characterized by marked differences in electrostatic potentials near the active site region compared to their mesophilic or thermophilic counterparts that may facilitate interaction with ligand. In the case of malate dehydrogenase, for example, the increased positive potential at and around the oxaloacetate binding site and the significantly decreased negative surface potential at the NADH binding region may facilitate the interaction of the oppositely charged ligands with the surface of the enzyme [131]. In all cases, the differences were caused by discrete substitutions in nonconserved charged residues resulting in local electrostatic potential differing in both sign and magnitude. Involvement of electrostatic potentials in cold-activity was also supported by a mutational study [178].

Finally, some unsuspected strategies have been shown to improve the activity in psychrophilic enzymes. With few exceptions, *β*-galactosidases are homotetrameric enzymes bearing four active sites. However, the crystal structure of a cold-active *β*-galactosidase revealed that it is a homohexamer, therefore possessing six active sites [125]. This unusual quaternary structure, containing two additional active sites, certainly contributes to improve the activity at low temperatures. Cellulases are microbial enzymes displaying a modular organization made of a globular catalytic domain connected by a linker to a cellulose binding domain. Psychrophilic cellulases were found to possess unusually long linkers of more than 100 amino acid residues, that is, about five times longer than in mesophilic cellulases [119, 179]. It was shown that the long linker adopts a large number of conformations between the fully extended and bended conformations, in caterpillar-like motions [119] and it was calculated that the catalytic domain has a 40-fold higher accessible surface area of substrate when compared with a mesophilic cellulase possessing a much shorter linker. Here also, increasing the surface of the insoluble substrate available to the catalytic domain should improve the activity of this enzyme at low temperatures.

### 4.3. Active Site Dynamics

The heat-labile activity of psychrophilic enzymes suggests that the dynamics of the functional side chains at the active site is improved in order to contribute to cold activity and the above-mentioned structural adaptations seem to favor a better accessibility to the substrate and release of the product. This view is strongly supported by the enzymological properties of cold-active enzymes. Nonspecific psychrophilic enzymes accept various substrates and have a broader specificity than the mesophilic homologues, because substrates with slightly distinct conformations or sizes can fit and bind to the site. For instance, the observed differences in substrate specificity between Atlantic salmon and mammalian elastases have been interpreted to be based on a somewhat wider and deeper binding pocket for the cold-adapted elastase [176]. The broad specificity of a psychrophilic alcohol dehydrogenase that oxidizes large bulky alcohols was also assigned to a highly flexible active site [180]. This active site dynamics in solution was also well demonstrated by a psychrophilic *α*-amylase [181]. Being more flexible, the active site can accommodate easily macromolecular polysaccharides and is more active on these substrates than a mesophilic homologue. By contrast, the flexible active site accommodates less efficiently short oligosaccharides and is less active on these substrates. Furthermore, the inhibition pattern of the psychrophilic *α*-amylase indicates that it can form a ternary complex (enzyme, substrate, and inhibitor), whereas the more rigid mesophilic homologue can only form binary complexes (enzyme and substrate or inhibitor). Several crystal structures of psychrophilic enzymes also point to an increased flexibility at or near the active site, as also supported by molecular dynamic simulations. 

### 4.4. Adaptive Drift and Adaptive Optimization of Substrate Affinity

As a consequence of the improved active site dynamics in cold-active enzymes, substrates bind less firmly in the binding site (if no point mutations have occurred) giving rise to higher *K*_*m*_ values. An example is given in Table 2 showing that a psychrophilic *α*-amylase is more active on its macromolecular substrates, whereas the *K*_*m*_ values are up to 30-fold larger; that is, the affinity for the substrates is up to 30-fold lower. Ideally, a functional adaptation to cold would mean optimizing both *k*_cat_ and *K*_*m*_. However, a survey of the available data on psychrophilic enzymes indicates that optimization of the *k*_cat_/*K*_*m*_ ratio is far from a general rule but on the contrary that the majority of cold-active enzymes improve the *k*_cat_ value at the expense of *K*_*m*_, therefore leading to suboptimal values of the *k*_cat_/*K*_*m*_ ratio, as also shown in Table 2. For instance, high *K*_*m*_ values have been reported for psychrophilic aspartate carbamoyltransferase [182, 183], triose-phosphate isomerase [137], subtilisin [184], lactate dehydrogénase [147, 162], DNA ligase [185], elongation factor Tu [186], glutamate dehydrogenase [187, 188], *α*-amylase [189], dihydrofolate reductase [190, 191], cellulase [179], endonuclease I [192], aspartate aminotransferase [193], isocitrate dehydrogenase [194], xylanase [195], ornithine carbamoyltransferase [196], citrate synthase [197], purine nucleoside phosphorylase [198], DEAD-Box proteins [99], and acetate kinase [199]. There is in fact an evolutionary pressure on *K*_*m*_ to increase in order to maximize the overall reaction rate. Such adaptive drift of *K*_*m*_ has been well illustrated by the lactate dehydrogenases from Antarctic fish [162] and by the psychrophilic *α*-amylase [189]. 

Several enzymes, especially in some cold-adapted fish, counteract this adaptive drift of *K*_*m*_ in order to maintain or to improve the substrate binding affinity by amino acid substitutions within the active site [176, 200]. The first reason for these enzymes to react against the drift is obvious when considering the regulatory function associated with *K*_*m*_, especially for intracellular enzymes. The second reason is related to the temperature dependence of weak interactions. Substrate binding is an especially temperature-sensitive step because both the binding geometry and interactions between binding site and ligand are governed by weak interactions having sometimes opposite temperature dependencies. Hydrophobic interactions form endothermically and are weakened by a decrease in temperature. By contrast, interactions of electrostatic nature (ion pairs, hydrogen bounds, and Van der Waals interactions) form exothermically and are stabilized at low temperatures. Therefore, low temperatures do not only reduce the enzyme activity (*k*_cat_), but can also severely alter the substrate binding mode according to the type of interaction involved. A comprehensive example was provided by a cold-active chitobiase: substitutions in the substrate binding site tend to replace hydrophobic residues (found in mesophilic chitobiases) by polar residues that are able to perform stronger interactions at low temperature [165].

### 4.5. Energetics of Cold Activity

**Figure 7:**
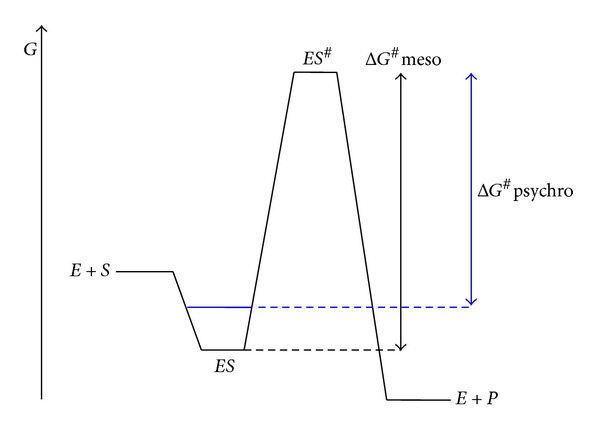
Optimization of activity by decreasing substrate affinity in psychrophilic enzymes. Reaction profile for an enzyme-catalyzed reaction with Gibbs energy changes under saturating substrate concentration. Weak substrate binding (in blue) decreases the activation energy (Δ*G* ^#^ *psychro*) and thereby increases the reaction rate. Adapted from [202].

Referring to (1), the high activity of cold-adapted enzymes corresponds to a decrease of the free energy of activation Δ*G* ^#^. Two strategies have been highlighted to reduce the height of this energy barrier.  Figure 7 illustrates the first strategy where an evolutionary pressure increases *K*_*m*_ in order to maximize the reaction rate. According to the transition state theory, when the enzyme encounters its substrate, the enzyme-substrate complex ES falls into an energy pit. For the reaction to proceed, an activated state ES^#^ has to be reached, that eventually breaks down into the enzyme and the product. The height of the energy barrier between the ground state ES and the transition state ES^#^ is defined as the free energy of activation Δ*G* ^#^: the lower this barrier, the higher the activity as reflected in (1). In the case of cold active enzymes displaying a weak affinity for the substrate, the energy pit for the ES complex is less deep (dashed in Figure 7). It follows that the magnitude of the energy barrier is reduced and therefore the activity is increased. This thermodynamic link between affinity and activity is valid for most enzymes (extremophilic or not) under saturating substrate concentrations and this link appears to be involved in the improvement of activity at low temperatures in numerous cold-active enzymes [162, 196, 201]. 

The second and more general strategy involves the temperature dependence of the reaction catalyzed by cold-active enzymes. The free energy of activation Δ*G* ^#^ contains both enthalpic and entropic terms according to the classical Gibbs-Helmholtz relation:

(2) $$\begin{array}{c} \Delta G^{\#}=\;\Delta H^{\#}-T\Delta S^{\#}. \end{array}$$

Accordingly, (1) can be rewritten as

(3) $$\begin{array}{c} k_{\text{cat}}=\frac{k_{B}T}{h}e^{-({\Delta H}^{\#}/RT-{\Delta S}^{\#}/R)}.\;\; \end{array}$$

Equation (3) shows that both Δ*H* ^#^ and Δ*S* ^#^ have the potential to alter *k*_cat_.  Table 3 reports the enthalpic and entropic contributions to the free energy of activation in extremophilic *α*-amylases. Similar data have been compiled for other psychrophilic enzymes [147, 158, 159, 163, 179, 192, 203, 204]. The enthalpy of activation Δ*H* ^#^ depicts the temperature dependence of the activity: the lower this value, the lower the variation of activity with temperature. The low value found for almost all psychrophilic enzymes demonstrates that their reaction rate is less reduced than for other enzymes when the temperature is lowered. Accordingly, the decrease of the activation enthalpy in the enzymatic reaction of psychrophilic enzymes can be considered as the main adaptive character to low temperatures [162, 201]. This decrease is structurally achieved by a decrease in the number of enthalpy-driven interactions that have to be broken during the activation steps. These interactions also contribute to the stability of the protein folded conformation and, as a corollary, the structural domain of the enzyme bearing the active site should be more flexible. It is interesting to note that such a macroscopic interpretation of the low activation enthalpy in cold-active enzymes fits with the experimental observation of a markedly heat-labile activity illustrated in Figure 5.  Table 3 shows that the entropic contribution *T*Δ*S* ^#^ for the cold-active enzyme is larger and negative. This has been interpreted as a large reduction of the apparent disorder between the ground state with its relatively loose conformation and the well-organized and compact transition state [163]. The heat-labile activity of cold-active enzymes suggests a macroscopic interpretation for this thermodynamic parameter. As a consequence of active site flexibility, the enzyme-substrate complex ES occupies a broader distribution of conformational states translated into increased entropy of this state, compared to that of the mesophilic or thermophilic homologues. This assumption has received strong experimental support [160, 205]. Furthermore, a broader distribution of the ground state ES should be accompanied by a weaker substrate binding strength, as indeed observed for numerous psychrophilic enzymes. Finally, it should be mentioned that the typical activation parameters of psychrophilic enzymes are well reproduced by reaction kinetic simulations [170].

## 5. Global and Local Dynamics

The previous sections have highlighted the recurrent involvement of dynamics or flexibility in cold-activity of psychrophilic enzymes. Determination of molecular flexibility is complex as it requires the definition of the types and amplitudes of atomic motions as well as a timescale for these motions [206–208]. Nevertheless, various biophysical studies have revealed a less compact conformation of psychrophilic enzymes, undergoing frequent microunfolding events.

**Figure 8:**
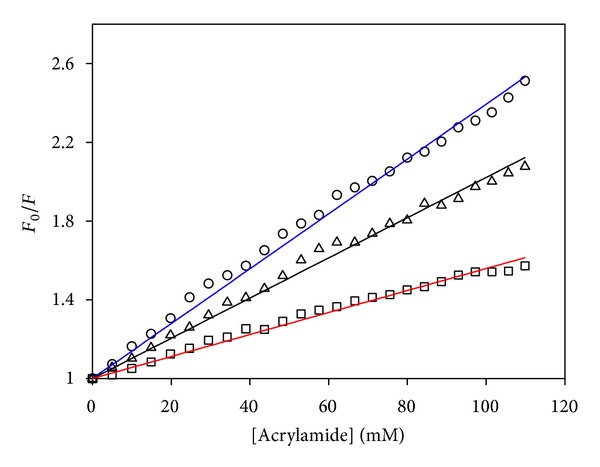
Permeability of the protein structure at room temperature. Fluorescence quenching experiments on psychrophilic (circles, blue) mesophilic (triangles, black), and thermophilic (squares, red) proteins. The steep slope recorded for the psychrophilic protein indicates that its structure is easily penetrated by a small quencher molecule (acrylamide), resulting in a larger attenuation of the intrinsic fluorescence (*F*_0_/*F*), whereas the thermophilic protein is more rigid and displays fewer internal motions. Adapted from [160].

In this respect, the first experimental approach is apparently the pioneering work of Privalov [209] reporting an extensive analysis of collagen structure from organisms adapted to different temperatures (including Antarctic and Arctic fish) and using kinetics of hydrogen/deuterium exchange to quantify flexibility. The author concluded that the correlation between physiological temperature and collagen stability can be explained by a definite level of motility of protein structure required for its efficient functioning under physiological temperatures. This suggests that adjustment of conformational flexibility is a key event in the thermal adaptation of proteins [210–212]. Such differences in flexibility have received further support by the quantification of macromolecular dynamics in the whole protein content of psychrophilic, mesophilic, thermophilic, and hyperthermophilic bacteria by neutron scattering [213]. Neutron spectroscopy provides a unique tool to study thermal atomic motions in macromolecules because neutron wavelengths and energies match motion amplitudes and frequencies, respectively [213, 214]. These experiments have indeed revealed that the resilience (equivalent to macromolecular rigidity in terms of a force constant) increases with physiological temperatures. Furthermore, it was also shown that the atomic fluctuation amplitudes (equivalent to macromolecular flexibility) were similar for each microorganism at its physiological temperature. This is in full agreement with Somero's “corresponding state” concept [215] postulating that protein homologues exhibit comparable flexibilities to perform their biological function at their physiologically relevant temperatures. Fluorescence quenching of extremophilic proteins was also used as this method averages most of the dynamic parameters into a single signal. This technique utilizes increasing concentrations of a small quencher molecule (acrylamide in Figure 8). The decrease of fluorescence arising from diffusive collisions between the quencher and protein fluorophores reflects the ability of the quencher to penetrate the structure and can be viewed as an index of protein permeability. It was found that the structure of a psychrophilic protein has an improved propensity to be penetrated by the small quencher molecule, whereas a thermophilic protein only displays moderate quenching effect. Accordingly, a psychrophilic protein is a flexible molecule displaying numerous opening and closing motions or frequent microunfolding events [158–160, 216].

Local flexibility, as opposed to global flexibility, was predicted to play a pivotal role in enzyme cold activity [162, 163]. This local flexibility was first suggested by the 3D structure of a cold-active trypsin [148] and later observed in stability studies of psychrophilic enzymes showing that, in some cases, the structural domain bearing the active site is less stable than the remaining of the protein [161, 165–167, 217]. This was further evidenced by hydrogen/deuterium exchange studies of a psychrophilic alcohol dehydrogenase [218]. It was shown that functional regions involved in substrate and cofactor binding exhibit greater flexibility compared to a thermophilic homologue and, furthermore, that local flexibility can be uncoupled from global thermal stability. This local flexibility was also correlated with activity parameters [164, 219–222]. Several X-ray structures of psychrophilic enzymes have also pointed to a local flexibility at or around the active site as well as in functional regions [120, 124, 130, 133, 139, 145, 147]. Furthermore, molecular dynamics simulations have frequently highlighted local flexibility as a strategy for cold adaptation [174, 222–232].

The requirement for either global or local flexibility is still under debate. Enzymes displaying global flexibility frequently process large substrates, presumably involving concerted motions of the whole molecules, whereas local flexibility is generally observed for enzymes acting on small substrates [57]. There are, however, too many exceptions to conclude definitively.

## 6. Conformational Stability of Psychrophilic Enzymes

The numerous insights for close relationships between activity and stability in psychrophilic enzymes have prompted investigations of their conformational stability in comparison with mesophilic and thermophilic counterparts. The low thermal stability of cold-adapted proteins, known for decades, has been highlighted by various techniques such as intrinsic fluorescence spectroscopy (Figure 5). However, the energetics of structure stability was essentially revealed by microcalorimetry [158–160, 189, 203, 216, 233, 234].

### 6.1. Microcalorimetric Studies

**Figure 9:**
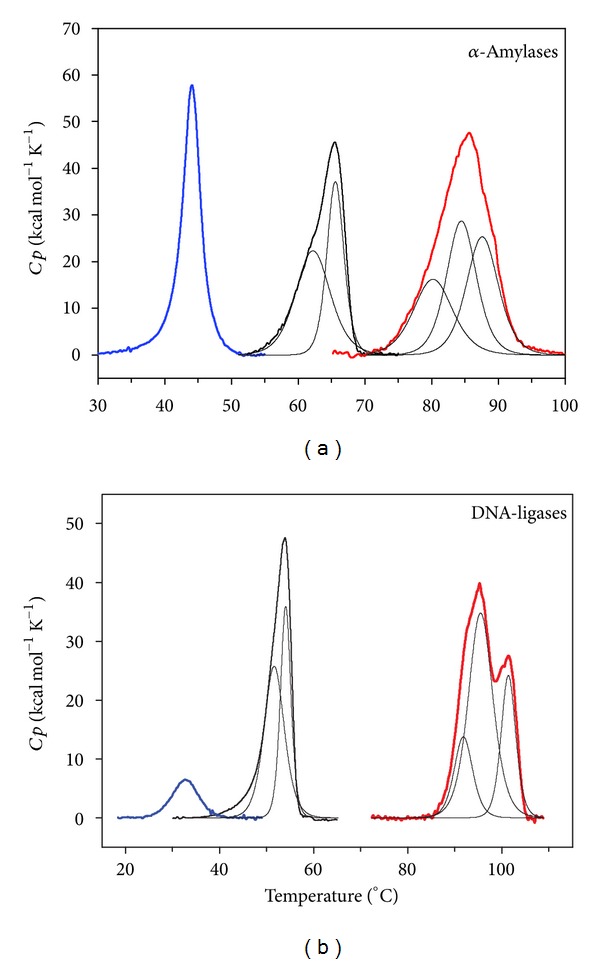
Thermal unfolding of extremophilic proteins. Thermograms of *α*-amylases and DNA-ligases recorded by differential scanning microcalorimetry showing, from left to right on each panel, psychrophilic (blue), mesophilic (black), and (hyper)thermophilic (red) proteins. The cold-adapted proteins are characterized by a lower *T*_*m*_ (top of the transition) and Δ*H*_cal_ (area under the transition), by a sharp and cooperative transition and by the lack of stability domains (indicated by thin lines in stable proteins). Adapted from [158, 189].

Microcalorimetric records of heat-induced unfolding for psychrophilic, mesophilic and thermophilic proteins are shown in Figure 9. These enzymes clearly show distinct stability patterns that evolve from a simple profile in the unstable psychrophilic proteins to a more complex profile in very stable thermophilic counterparts. The unfolding of the cold-adapted enzymes occurs at lower temperatures as indicated by the temperature of half-denaturation *T*_*m*_, given by the top of the transition. The calorimetric enthalpy Δ*H*_cal_ (area under the curves in Figure 9) corresponding to the total amount of heat absorbed during unfolding reflects the enthalpy of disruption of bonds involved in maintaining the compact structure and is markedly lower for the psychrophilic enzymes (Table 4). In addition, there is a clear trend for increasing Δ*H*_cal_ values in the order psychrophile < mesophile < thermophile. The transition for the psychrophilic enzymes is sharp and symmetric, whereas other enzymes are characterized by a flattening of the thermograms. This indicates a pronounced cooperativity during unfolding of the psychrophilic enzymes: the structure is stabilized by fewer weak interactions and disruption of some of these interactions strongly influences the whole molecular edifice and promotes its unfolding. The psychrophilic enzymes unfold according to an all-or-none process, revealing a uniformly low stability of the architecture. By contrast, all other homologous enzymes display two to three transitions (indicated by deconvolution of the heat capacity function in Figure 9). Therefore, the conformation of these mesophilic and thermophilic enzymes contains structural blocks or units of distinct stability that unfold independently. Finally, the unfolding of the psychrophilic proteins is frequently more reversible than that of other homologous enzymes that are irreversibly unfolded after heating. The weak hydrophobicity of the core clusters in cold-adapted enzymes and the low temperature for unfolding, which prevent aggregation, certainly account for this reversible character.

### 6.2. Stability Curves

The thermodynamic stability of a protein that unfolds reversibly according to a two-state mechanism is described by the classical Gibbs-Helmholtz relation:

(4) $$\begin{array}{c} \Delta G_{N-U}=\Delta H_{N-U}-T\Delta S_{N-U}. \end{array}$$

**Figure 10:**
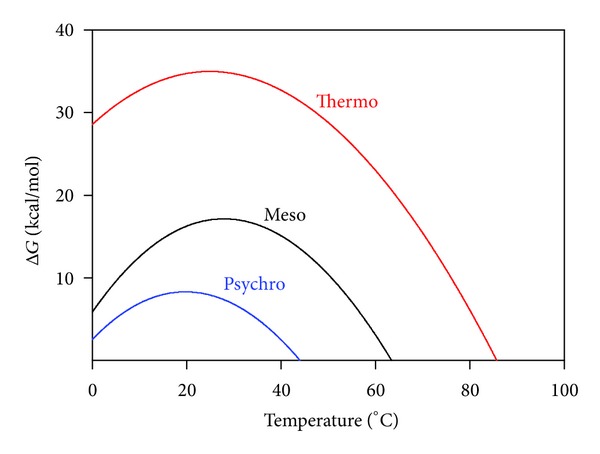
Gibbs free energy of unfolding, or conformational stability, of homologous extremophilic proteins. The work required to disrupt the native state is plotted as a function of temperature. The high stability of the thermophilic protein is reached by lifting the curve towards higher free energy values, whereas the low stability of the psychrophilic proteins corresponds to a global collapse of the bell-shaped stability curve. Adapted from [160].

The latter relation can be rewritten for any temperature (*T*) using the parameters determined experimentally by DSC:

(5) ΔGN−U(T)  =  ΔHcal(1−TTm)  +  ΔCp(T−Tm)−  TΔCp  ln⁡⁡(TTm),

where Δ*Cp* is the difference in heat capacity between the native and the unfolded states. This parameter reflects the hydration of nonpolar groups that are exposed to water upon unfolding. Computing (5) in a temperature range where the native state prevails provides the protein stability curve [235], that is, the free energy of unfolding as a function of temperature (Figure 10). In other words, this is the work required to disrupt the native state at any given temperature [236] and is also referred to as the thermodynamic stability. By definition, this stability is zero at *T*_*m*_ (equilibrium constant *K* = [*U*]/[*N*] = 1 and Δ*G* = − *RT*ln⁡⁡*K*). At temperatures below *T*_*m*_, the stability increases, as expected, but perhaps surprisingly for the nonspecialist, the stability reaches a maximum close to room temperature then it decreases at lower temperatures (Figure 10). In fact, this function predicts a temperature of cold unfolding, which is generally not observed because it occurs below 0°C [237]. Increasing the stability of a protein is essentially obtained by lifting the curve towards higher free energy values [238], whereas the low stability of a psychrophilic protein is reached by a global collapse of its curve. As far as extremophiles are concerned, one of the most puzzling observations of the last decade is that most proteins obey this pattern; that is, whatever the microbial source is, the maximal stability of their proteins is clustered around room temperature [238–240], although exceptions have been reported [241]. Accordingly, the environmental temperatures for mesophiles and thermophiles lie on the right limb of the bell-shaped stability curve and obviously, the thermal dissipative force is used to promote molecular motions in these molecules. By contrast, the environmental temperatures for psychrophiles lie on the left limb of the stability curve. It follows that molecular motions in proteins at low temperatures are gained from the factors ultimately leading to cold unfolding [233], that is, the hydration of polar and nonpolar groups [242]. The origin of flexibility in psychrophilic enzymes at low temperatures is therefore drastically different from mesophilic and thermophilic proteins, the latter taking advantage of the conformational entropy rise with temperature to gain in mobility. 

A surprising consequence of the free energy function for the psychrophilic protein shown in Figure 10 is its weak stability at low temperatures when compared with mesophilic and thermophilic proteins, whereas it was intuitively expected that cold-active proteins should also be cold stable. This protein is in fact both heat and cold labiles. Therefore cold denaturation of some key enzymes in psychrophiles can be an additional, though unsuspected factor fixing the lower limit of life at low temperatures. 

### 6.3. Structural Origin of Low Stability

**Figure 11:**
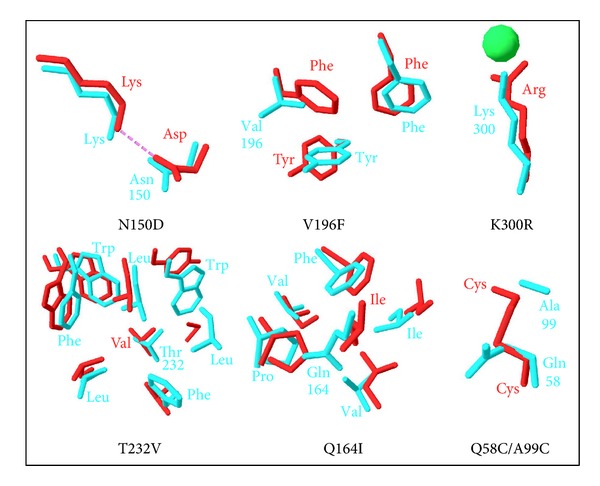
Amino acid substitutions in a psychrophilic alpha-amylase (blue) in comparison with the structure of its mesophilic homologue (red). The substitution N150D disrupts a salt bridge with the corresponding Lys side chain in the cold-adapted protein. The substitution V196F disrupts a triple face-to-edge aromatic interaction. Substitution K300R results in a monodentate coordination of the chloride ion, instead of a bidentate coordination by Arg in the mesophilic protein, as demonstrated by the crystal structure of the single mutant [272]. Substitutions T232V and Q164I decrease the apolarity of hydrophobic core clusters in the psychrophilic enzyme and the double substitution Q58C/A99C eliminates a disulfide bond. Adapted from [114].

Most of X-ray crystal structures from psychrophilic enzymes listed in Table 1 have been analyzed in order to decipher the structural origins of both cold activity and weak stability. However, the interpretation of these data is frequently difficult because these structural adaptations are extremely discrete and can easily escape the analysis, as exemplified in Figure 11. For instance, it has been shown that difference in distance interaction between enzyme and substrate as low as 0.1 Å (i.e., well below of the resolution of most crystal structures) can lead to substantial difference in catalytic efficiency [243]. Furthermore, these structural adaptations are very diverse, reflecting the complexity of factors involved in the stability of a macromolecule at the atomic level. As a general picture, it was found that all structural factors currently known to stabilize the protein molecule could be attenuated in strength and number in the structure of cold-active enzymes [176, 244–247]. Two review articles can be consulted for a comprehensive discussion of this topic [108, 176].

The determinants of protein stability include structural factors, hydrophobic effects, and mainly weak interactions between atoms of the protein structure. In psychrophilic proteins, this involves the clustering of glycine residues, providing local mobility [248, 249]; the disappearance of proline residues in loops, enhancing chain flexibility between secondary structures [250]; a reduction in arginine residues which are capable of forming multiple salt bridges and H-bonds [251] as well as a lower number of ion pairs, aromatic interactions, or H-bonds, compared to mesophilic enzymes [148, 252]. The size and relative hydrophobicity of nonpolar residue clusters forming the protein core is frequently smaller, lowering the compactness of the protein interior by weakening the hydrophobic effect on folding [253]. Psychrophilic proteins have larger cavity sizes sufficient to accommodate water molecules: these cavities and embedded water molecules can play a significant role in structural flexibility [254]. The *N*- and *C*-caps of *α*-helices are also altered, weakening the charge-dipole interaction [255] and loose or relaxed protein extremities appear to be preferential sites for unzipping [148, 256]. The binding of stabilizing ions, such as calcium, can be extremely weak, with binding constants differing from mesophiles by several orders of magnitude [157, 257]. Insertions and deletions are sometimes responsible for specific properties such as the acquisition of extrasurface charges via insertions [157] or the weakening of subunit interactions via deletions [255]. Occasionally, disulfide linkages are lacking in cold-adapted proteins [234, 257, 258].

Calculation of the solvent accessible area showed that some psychrophilic enzymes expose a higher proportion of nonpolar residues to the surrounding medium [116, 124, 158]. This is an entropy-driven destabilizing factor caused by the reorganization of water molecules around exposed hydrophobic side chains. Calculations of the electrostatic potential revealed in some instances an excess of negative charges at the surface of the protein and, indeed, the pI of cold-active enzymes is frequently more acidic than that of their mesophilic or thermophilic homologues. This has been related to improved interactions with the solvent, which could be of prime importance in the acquisition of flexibility near zero degrees [233]. Besides the balance of charges, the number of salt bridges covering the protein surface is also reduced. There is a clear correlation between surface ion pairs and temperature adaptation, since these electrostatic interactions significantly increase in number from psychrophiles to mesophiles, to thermophiles, and hyperthermophiles, the latter showing arginine-mediated multiple ion pairs and interconnected salt bridge networks [259, 260]. Such an altered pattern of electrostatic interactions is thought to improve the dynamics or the “breathing” of the external shell of cold-active enzymes.

However, each enzyme adopts its own strategy by using one or a combination of these altered structural factors in order to improve the local or global mobility of the protein edifice. Accordingly, a general theory for structural adaptations cannot be formulated but nevertheless, enzyme families sharing the 3D fold can be compared as reviewed in [231]. Comparative structural analyses of psychrophilic, mesophilic, and thermophilic enzymes indicate that each protein family displays different structural strategies to adapt to temperature [135, 231, 245, 261–266]. However, some common trends are observed: the number of ion pairs, the side-chain contribution to the exposed surface, and the apolar fraction of the buried surface show a consistent decrease with decreasing optimal temperatures [245]. The multitude of structural strategies in cold-adapted proteins has also complicated statistical analyses aimed at delineating general trends in temperature adaptation. Various trends have been reported for psychrophilic proteins using different methodologies and datasets: a preference for smaller-size and less hydrophobic residues [267]; a less hydrophobic core, less charged, and long-chained surface residues [268]; a decrease of solvent accessible surface contribution of charged residues and an increase of hydrophobic surface contribution [37] or various patterns of preferential amino acid substitutions [269–271]. As a result of the diversity of structural factors involved in cold adaptation, the mean amino acid composition of whole genomes does not provide clear trends of preferential residue utilization [37–40].

## 7. Activity-Flexibility-Stability Relationships: Current Issues

### 7.1. Experimental Insights

**Figure 12:**
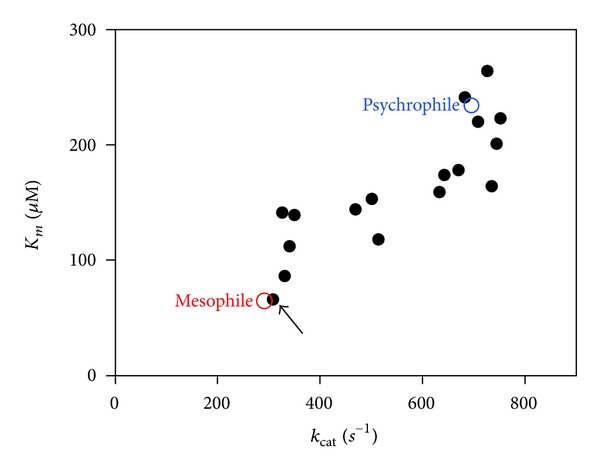
Engineering mesophilic-like activity in mutants of a psychrophilic *α*-amylase. This plot of the kinetic parameters for the stabilized mutants (filled symbols) shows that the general trend is to decrease the activity and to increase the affinity for the substrate of the wild-type psychrophilic enzyme (open symbol). The most stable mutant bearing six additional interactions (arrow) displays kinetic parameters nearly identical to those of the mesophilic homologue (open symbol). Adapted from [189, 273].

In order to check the validity of the proposed relationships between the activity and the stability in cold-active enzymes, a psychrophilic *α*-amylase has been used as a model because the identical architecture of its active site, when compared with a close mesophilic homologue (Figure 6), indicates that structural adaptations affecting the active site properties occur outside from the catalytic cavity. On this basis, 17 mutants of this enzyme were constructed, each of them bearing an engineered residue forming a weak interaction found in mesophilic *α*-amylases but absent in the cold-active *α*-amylase as well as combinations of up to six stabilizing structural factors [114, 189, 234, 273]. It was shown that these engineered interactions improve all the investigated parameters related to protein stability: the compactness, the kinetically driven stability, the thermodynamic stability, the resistance towards chemical denaturation, and the kinetics of unfolding/refolding.Therefore, these mutants of the psychrophilic *α*-amylase consistently approximate and reproduce the stability and unfolding patterns of the heat-stable enzymes. These effects were also analyzed by molecular dynamics [274]. Concomitantly to this improved stability, stabilized mutants have lost the kinetic optimization to low temperature activity displayed by the parent psychrophilic enzyme. As shown in Figure 12, stabilizing the cold-active *α*-amylase tends to decrease the *k*_cat_ values and concomitantly the *K*_*m*_ values of the mutant enzymes, revealing the high correlation between both kinetic parameters. In fact, in addition to an engineered mesophilic-like stability, the multiple-mutant bearing six stabilizing structural factors also displays an engineered mesophilic-like activity in terms of alterations in *k*_cat_ and *K*_*m*_ values and even in thermodynamic parameters of activation [114, 273]. These results provide strong experimental support to the hypothesis assuming that the disappearance of stabilizing interactions in psychrophilic enzymes increases the amplitude of concerted motions required by catalysis and the dynamics of active site residues at low temperature, leading to a higher activity. 

It should be mentioned that other mutational studies of cold-active enzymes have been less conclusive. Various psychrophilic enzymes [178, 184, 248–250, 275–282] have been engineered in order to check the involvement of specific adaptive mutations within or close to the active site. These studies have revealed a complex pattern of effects on kinetic parameters, activation energy, and stability that cannot be interpreted in simple terms.

### 7.2. Directed Evolution

The activity-stability relationships in cold-adapted proteins have been challenged from an evolutionary point of view. As a matter of fact, directed evolution [283, 284] and protein engineering [285] of enzymes have demonstrated that activity and stability are not physically linked in protein, as reviewed in [286]. Accordingly, it has been proposed that the low stability of cold-active enzymes is the result of a genetic drift related to the lack of selective pressure for stable proteins. Although this seems to be obvious, several lines of evidence indicate that the situation is more subtle. As already mentioned, in multidomain psychrophilic enzymes, the catalytic domain is always heat labile, whereas the noncatalytic domain can be as stable as mesophilic proteins [165–167]. It is therefore unlikely that a genetic drift only affects the catalytic domain without modifying other regions of the protein. Furthermore, several directed evolution experiments have shown that when libraries of randomly mutated enzymes are only screened for improved activity at low temperatures without any other constraints, the best candidates invariably display the canonical properties of psychrophilic enzymes, as discussed in [287]. Examination of the activity versus stability plots for hundreds of mutants shows that random mutations improving both activity and stability are rare [288, 289]. It follows that improvement of activity at low temperatures associated with loss of stability appears to be the most frequent and accessible event. In conclusion, the current view suggests that the strong evolutionary pressure on psychrophilic enzymes to increase their activity at low temperatures can be accommodated for by the lack of selection for stability and represents the simplest adaptive strategy for enzyme catalysis in the cold.

### 7.3. Noticeable Exceptions

Considering the above-mentioned lessons from laboratory evolution, one should expect that psychrophilic enzymes, which do not share the canonical properties, can be discovered. Indeed, some reports suggest the occurrence of noticeable exceptions to the general rule. For instance, single-point mutations can account for most of the adaptive traits. A single mutation in the calcium-binding site of a psychrophilic subtilisin [184] and in the phosphate-binding helix of triose phosphate isomerase [137] increases drastically the thermostability of the psychrophilic mutants. Conversely, a single amino acid substitution near the active site of a thermostable *α*-glucosidase provides cold activity [290]. The chaperonin and heat-shock protein GroEL from an Antarctic bacterium is not cold adapted and displays similar stability and activity than that of its *E. coli* homologue [291]. It has been suggested that this chaperonin remains suited to function during sudden temperature increases of the environment [83]. Similarly, activity of thioredoxin from the same bacterium is much more heat stable than that of *E. coli* [292, 293]. One cannot exclude that enzymes involved in electron transfer do not require the same type of adaptations because the rate of electron flow is not significantly affected by the low biological temperatures. Isocitrate dehydrogenase from a psychrophilic bacterium is more stable than its mesophilic homologue, while cold activity was attributed to local flexibility at the active site [133]. 

## 8. Folding Funnel Model for Psychrophilic Enzymes

**Figure 13:**
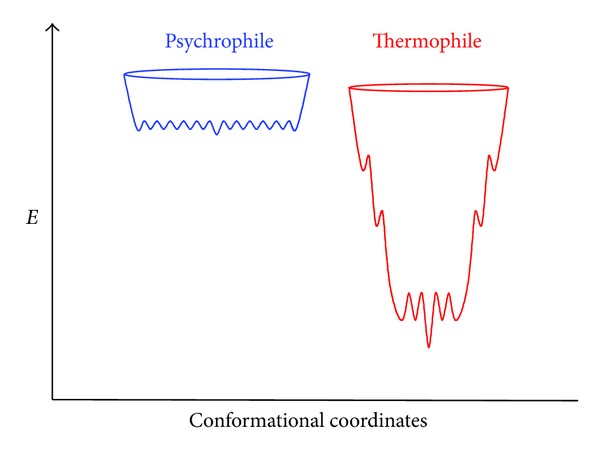
Folding funnel model of enzyme temperature adaptation. In these schematic energy landscapes for extremophilic enzymes, the free energy of folding (*E*) is depicted as a function of the conformational diversity. The height of the funnels is deduced from the determination of the conformational stabilities. The top of the funnels is occupied by the unfolded states in the numerous random coil conformations, whereas the bottom of the funnels corresponds to native and catalytically active conformations. The ruggedness of the bottom depicts the energy barriers for interconversion, or structural fluctuations of the native state [160].

The various properties of psychrophilic enzymes have been integrated in a model based on folding funnels [294, 295] to describe the activity-stability relationships in extremophilic enzymes [160]. The energy landscapes of psychrophilic and thermophilic enzymes are shown in Figure 13. The top of the funnel is occupied by the unfolded state having a high free energy (considering the spontaneous folding reaction), whereas the bottom of the funnel is occupied by the stable (low free energy) native state. The height of the funnel, that is, the free energy of folding, also corresponding to the conformational stability, has been fixed here in a 1 to 5 ratio (Figure 10). The upper edge of the funnels is occupied by the unfolded state in random coil conformations but it should be noted that psychrophilic enzymes tend to have a lower proline content than mesophilic and thermophilic enzymes, a lower number of disulfide bonds and a higher occurrence of glycine clusters. Accordingly, the edge of the funnel for the psychrophilic protein is slightly larger (broader distribution of the unfolded state) and is located at a higher energy level. When the polypeptide is allowed to fold, the free energy level decreases as well as the conformational ensemble. However, thermophilic proteins pass through intermediate states corresponding to local minima of energy. These minima are responsible for the ruggedness of the funnel slopes and for the reduced cooperativity of the folding-unfolding reaction, as demonstrated by heat-induced unfolding (Figure 9). By contrast, the structural elements of psychrophilic proteins generally unfold cooperatively without intermediates, as a result of fewer stabilizing interactions and stability domains; therefore, the funnel slopes are steep and smooth. The bottom of the funnel depicts the stability of the native state ensemble. The bottom for a very stable and rigid thermophilic protein can be depicted as a single global minimum or as having only a few minima with high energy barriers between them, whereas the bottom for an unstable and flexible psychrophilic protein is rugged and depicts a large population of conformers with low-energy barriers to flip between them. Rigidity of the native state is therefore a direct function of the energy barrier height [296, 297] and is drawn here according to a global flexibility of cold-adapted proteins. In this context, the activity-stability relationships in these extremophilic enzymes depend on the bottom properties. Indeed, it has been argued that upon substrate binding to the association-competent subpopulation, the equilibrium between all conformers is shifted towards this subpopulation, leading to the active conformational ensemble [296–299]. In the case of the rugged bottom of psychrophilic enzymes, this equilibrium shift only requires a modest free energy change (low energy barriers), a low enthalpy change for interconversion of the conformations but is accompanied by a large entropy change for fluctuations between the wide conformer ensemble. The converse picture holds for thermophilic enzymes, in agreement with the activation parameters shown in Table 3 and with the proposed macroscopic interpretation. Such energy landscapes integrate nearly all biochemical and biophysical data currently available for extremophilic enzymes but they will certainly be refined by future investigations of other series of homologous proteins from psychrophiles, mesophiles, and thermophiles. This model has nevertheless received support from several experimental and computational studies [170, 205, 207, 252, 299–302].

## 9. Psychrophilic Enzymes in Biotechnology

As already mentioned, most enzymes from psychrophiles are cold active and heat labile. These specific traits are responsible for the three main advantages of cold active enzymes in biotechnology: (i) as a result of their high activity, a lower concentration of the enzyme catalyst is required to reach a given activity, therefore reducing the amount of costly enzyme preparation in a process; (ii) as a result of their cold activity, they remain efficient at tap water or ambient temperature, therefore avoiding heating during a process, either at domestic (e.g., washing machine) or industrial levels; and (iii) as a result of heat lability, they can be efficiently and sometime selectively inactivated after a process by moderate heat input. Beside these traits, enzymes from organisms endemic to cold environments can be a valuable source of new catalysts possessing useful enzymological characteristics such as novel substrate specificities or product properties. Previous reviews should be consulted for a complete coverage of this topic [16, 28, 303–308]. Bioprospector, an online database (http://www.bioprospector.org/bioprospector/) provides a survey of patents, commercial products, and companies involved in applied research using genetic resources from both the Antarctic and the Arctic. Some specific examples are provided below.

### 9.1. Heat Lability in Molecular Biology

Alkaline phosphatases are mainly used in molecular biology for the dephosphorylation of DNA vectors prior to cloning to prevent recircularization or for the dephosphorylation of 5′-nucleic acid termini before 5′-end labeling by polynucleotide kinase. However, the phosphatase has to be carefully removed after dephosphorylation to avoid interferences with the subsequent steps. It follows that heat-labile alkaline phosphatases are excellent alternatives as they are inactivated by moderate heat treatment allowing to perform the subsequent steps in the same test tube and minimizing nucleic acid losses [309]. An alkaline phosphatase from an Antarctic bacterium has been fully characterized [128, 310, 311]. This heat-labile alkaline phosphatase, sold as Antarctic phosphatase, is now proposed on the market by New England Biolabs Inc. (Ipswich, MA, USA). In the same context, the heat-labile alkaline phosphatase from the Arctic shrimp *Pandalus borealis* is also available for instance from Biotec Pharmacon ASA (Tromsø, Norway) or GE Healthcare Life Sciences (Little Chalfont, UK).

Two other psychrophilic enzymes are also marketed for molecular biology applications taking advantage of the heat-labile property. Shrimp nuclease selectively degrades double-stranded DNA: for instance, it is used for the removal of carry-over contaminants in PCR mixtures, and then it is heat inactivated prior addition of the template. This recombinant enzyme is available from Biotec Pharmacon ASA (Tromsø, Norway), USB Corporation (Santa Clara, CA, USA), or Thermo Scientific (Waltham, MA, USA). Heat-labile uracil-DNA *N*-glycosylase from Atlantic cod (*Gadus morhua*), that presents typical cold adaptation features [151], is also used to remove DNA contaminants in sequential PCR reactions. Following degradation of contaminants, the enzyme is completely and irreversibly inactivated after heat treatment. Heat-labile uracil-DNA *N*-glycosylase is available from Biotec Pharmacon ASA (Tromsø, Norway).

### 9.2. Low Temperature Activity

The market for enzymes used in detergents represents 30%–40% of all enzymes produced worldwide. Amongst these enzymatic cleaning agents, subtilisin (an alkaline serine protease predominantly produced by *Bacillus* species) largely dominates this market. At the domestic level, the current trend is, however, to use detergents at lower washing temperatures because of the associated reductions in energy consumption and costs as well as to protect texture and colors of the fabrics. Accordingly, cold-active subtilisins are required for optimal washing results at tap water temperatures and the current advertisements for cold-active detergents indicate that this goal has been reached. The first psychrophilic subtilisins isolated from Antarctic *Bacillus* species have been extensively characterized to comply with such requirements [157, 184]. Subtilisins currently incorporated in cold-active detergents are engineered enzymes that combine storage stability, alkaline stability, and activity and cold activity. Although psychrophilic subtilisins are not components *per se* of cold-active detergents, they have largely contributed to the advancement of this economically attractive concept.

Beta-galactosidase, or lactase, is a glycoside hydrolase that specifically hydrolyzes the milk sugar lactose into galactose and glucose. It should be stressed that 75% of the world population suffers from lactose intolerance arising from deficient synthesis of intestinal lactase in adults and resulting in digestive disorders. In this context, a cold-active lactase from an Antarctic bacterium has been patented (WO 01/04276A1) for its capacity to hydrolyze lactose during milk storage at low temperatures [204]. This cold-active lactase will be also produced soon in large quantities by Nutrilab NV (Bekkevoort, Belgium) to hydrolyze lactose (as a by-product of the dairy industry) in the process of the high value sweetener D-tagatose, a natural monosaccharide with low caloric value and glycemic index.

Protein chaperones assist the folding of nascent polypeptides, preventing misfolding or even repairing misfolding. Taking advantage of these properties, the ArcticExpress *E. coli* cells from Stratagene (USA) have been engineered to coexpress cold-active chaperonins from an Antarctic bacterium [102] with the recombinant protein of interest, therefore improving protein processing at low temperatures (that prevent inclusion bodies formation) and increasing the yield of active, soluble recombinant protein.

### 9.3. New Specificities in Psychrophilic Enzymes

At the industrial level, the yeast *Candida antarctica* produces two lipases, A and B, the latter being sold for instance as Novozym 435 by Novozymes (Bagsvaerd, Denmark). As a result of its substrate and stereospecificity, lipase B is involved in a very large number of organosynthesis applications related to food/feed processing, pharmaceuticals, or cosmetics [312]. In a survey of patents related to Antarctica [313], it was shown that lipases from *C. antarctica* by far dominate the number of process- or product-based patents. This is a significant example of the potential for novel catalysts from genetic resources in cold environments.

**Figure 14:**
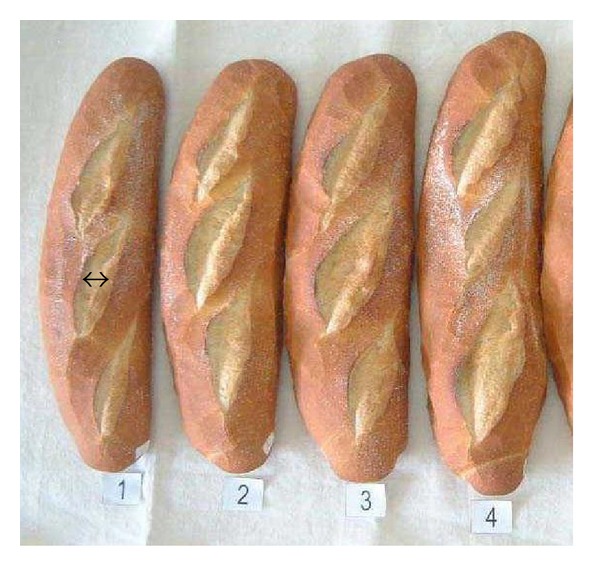
Effects of xylanases on bread volume and cut width (arrows) as compared to a negative control without added xylanase (loaf 1). Adapted from [315].

Xylanases are glycoside hydrolases that degrade the polysaccharide beta-1,4-xylan, thus breaking down hemicellulose, one of the major components of plant cell walls. Xylanases are a key ingredient of industrial dough conditioners used to improve bread quality. It was found that the xylanase from an Antarctic bacterium belongs to a new class of xylanases [118, 159, 171, 195, 314]. Furthermore, baking trials have revealed that the psychrophilic xylanase was very effective in improving the dough properties and final bread quality with, for instance, a positive effect on loaf volume, as shown in Figure 14 [315, 316]. This efficiency appears to be related to the high activity of the psychrophilic xylanase at cool temperatures required for dough resting and to its specific mode of xylan hydrolysis. This xylanase is now sold by Puratos (Grand-Bigard, Belgium). This is apparently the psychrophilic enzyme produced at the highest amounts to date.

### 9.4. Other Psychrophilic Proteins in Biotechnology

In this context, some cold-adapted proteins are also worth mentioning. This includes a recombinant antifreeze protein from an Arctic fish added in several edible ice cream brands from Unilever (The Netherlands, England) for its ice-structuring properties; the bacterial antifreeze Antarticine-NF3 (sometimes under the name Antarctilyne), which is effective for scar treatments and reepithelialization of wounds, or extracts of the Antarctic algae *Durvillea antarctica*, which are included in cosmetics to improve skin vitality such as in the Extra Firming Day Cream, a top seller of Clarins (France). Improved cold activity by chemical modification of mesophilic enzymes has also been reported [317–319] and was proven successful in some specific cases.

## 10. Conclusions

Psychrophiles thriving permanently at near-zero temperatures synthesize cold-active enzymes to sustain their cell cycle. Most psychrophilic enzymes optimize a high activity at low temperature at the expense of substrate affinity, therefore reducing the free energy barrier of the transition state. Furthermore, a weak temperature dependence of activity ensures moderate reduction of the catalytic activity in the cold. In these naturally evolved enzymes, the optimization to low temperature activity is reached via destabilization of the structures bearing the active site or by destabilization of the whole molecule. This involves a reduction in the number and strength of all types of weak interactions or the disappearance of stability factors, resulting in improved dynamics of active site residues in the cold. These enzymes are already used in many biotechnological applications requiring high activity at mild temperatures or fast heat-inactivation rate.

## 11. Future Avenues

Although significant progresses have been recently achieved in the understanding of protein adaptation to extreme environmental temperatures, many questions remain to be addressed with regards to structure-function relationships in psychrophilic proteins. In a previous review on cold-active enzymes [108], various open questions have been also highlighted and this reference should be consulted for the sake of completeness.

*Folding at Low Temperature. *The folding reaction of a protein should be strongly dependent of the temperature at which any organism synthesizes the polypeptide: how are these polypeptides synthesized in the cold, is the amino acid sequence adapted to cold folding, what is the rate-limiting step of folding, and are cold-adapted chaperones involved?

*Kinetic Parameters. *Most cold-active enzymes display improved *k*_cat_ values at low temperature associated with increased (unfavorable) *K*_*m*_ values, whereas in some cases the *K*_*m*_ is improved. What are the selective pressure and physiological requirements for such adjustments in substrate binding? Which types of psychrophilic enzymes do require such adjustments? Surprisingly, no detailed enzymological analysis of psychrophilic enzymes has been reported, except one [200]. Accordingly, what are the fine adjustments of enzyme activity at low temperature?

*Global versus Local Flexibility. *Structural adjustments around the active site render the catalytic center more dynamic but in many cases the whole molecule is more dynamic. What are the requirements for local or global flexibility? In parallel with the previous question, some psychrophilic enzymes are uniformly unstable (structure and activity), whereas others only display heat-labile activity: what are the tradeoffs in activity and stability?

*Natural Selection. *To what extent the weak stability of psychrophilic enzymes is the result of the lack of selection for stable proteins or of the strong selection for high activity? What is the balance between both selective pressures? 

*Macromolecular Dynamics. *The improved dynamics of psychrophilic enzymes is a central theme of previous investigations but what are the most reliable techniques to investigate this aspect and how to discriminate between local and global dynamics? X-ray structures provide a static picture when comparing psychrophilic, mesophilic, or thermophilic proteins. More sophisticated and insightful methods are needed such as NMR, neutron scattering, and H/D exchanges.

*Folding Funnels. *The global model depicting the activity-flexibility-stability relationships in psychrophilic enzymes in Figure 13 is attractive but it was built on a limited number of case studies: can we generalize this model or are there some variations and refinements to this model?

*Extreme Environmental Temperatures. *How do psychrophilic proteins compare with thermophilic proteins? Are there specific adaptive mechanisms to either low or high temperatures? Or, is there a continuum in the adaptive strategies from low to moderate and high temperatures? 

It is the wish of the present author that such open questions will stimulate young researchers to embark in this field in order to refine our understanding of cold-adapted proteins and to contribute to this fascinating topic which encompass microbiology, enzymology, biochemistry, or biophysics. 

Note: The genome sequence of the Antarctic bacterium *Oleispira antarctica* has been recently deposited to GenBank, as well as the crystal structure of eleven proteins from this bacterium in the PDB. NMR assignments of a new psychrophilic protein have been also reported [320].

## Figures and Tables

**Table 1:** Representative psychrophilic enzymes of known crystal structure.

Cold-adapted protein	Source	PDB entry	References
Alpha-amylase	Antarctic bacterium *Pseudoalteromonas haloplanktis *	1AQH	[116, 117]
Xylanase	Antarctic bacterium *Pseudoalteromonas haloplanktis *	1H12	[118]
Cellulase	Antarctic bacterium *Pseudoalteromonas haloplanktis *	1TVN	[119]
Superoxide dismutase	Antarctic bacterium *Pseudoalteromonas haloplanktis *	3LJF	[120]
S-Formylglutathione hydrolase	Antarctic bacterium *Pseudoalteromonas haloplanktis *	3LS2	[121]
Ca^2+^ Zn^2+^ protease	Antarctic bacterium *Pseudomonas *sp.	1G9K	[122]
Beta-lactamase	Antarctic bacterium *Pseudomonas fluorescens *	2QZ6	[123]
Citrate synthase	Antarctic bacterium *Arthrobacter *sp.	1A59	[124]
Beta-galactosidase	Antarctic bacterium *Arthrobacter *sp.	1YQ2	[125]
Subtilisine	Antarctic bacterium *Bacillus *sp.	2GKO	[126]
Aliphatic amidase	Antarctic bacterium *Nesterenkonia *sp.	3HKX	[127]
Alkaline phosphatase	Antarctic bacterium	2IUC	[128]
Aminopeptidase	Arctic bacterium *Colwellia psychrerythraea *	3CIA	[129]
Phenylalanine hydroxylase	Arctic bacterium *Colwellia psychrerythraea *	2V27	[130]
Malate dehydrogenase	Arctic bacterium *Aquaspirillum arcticum *	1B8P	[131]
Serine proteinase	Arctic bacterium *Vibrio *sp.	1SH7	[132]
Isocitrate dehydrogenase	Arctic bacterium *Desulfotalea psychrophila *	2UXQ	[133]
Aspartate carbamoyltransferase	Deep-sea bacterium *Moritella profunda *	2BE7	[134]
Adenylate kinase	Bacteria *Bacillus globisporus *and *Marinibacillus marinus *	1S3G, 3FB4	[135, 136]
Triose-phosphate isomerase	Marine bacterium *Vibrio marinus *	1AW1	[137]
Ca^2+^ Zn^2+^ protease	Marine bacterium *Flavobacterium *sp.	1U1R	[138]
Tyrosine phosphatase	Bacterium *Shewanella *sp.	1V73	[139]
Catalase	Bacterium *Vibrio salmonicida *	2ISA	[140]
Endonuclease I	Bacterium *Vibrio salmonicida *	2PU3	[141]
Lipase	Bacterium *Photobacterium lipolytica *	2ORY	[142]
Superoxide dismutase	Bacterium *Aliivibrio salmonicida *	2W7W	[143]
Alkaline phosphatase	Bacteria *Vibrio *sp.* and Shewanella *sp.	3E2D, 3A52	[144, 145]
Alkaline phosphatase	Arctic shrimp *Pandalus borealis *	1K7H	[146]
Lactate dehydrogenase	Antarctic icefish *Champsocephalus gunnari *	2V65	[147]
Trypsin	Atlantic salmon *Salmo salar *	2TBS	[148]
Elastase	Atlantic salmon *Salmo salar *	1ELT	[149]
Pepsin	Atlantic cod *Gadus morhua *	1AM5	[150]
Uracil-DNA glycosylase	Atlantic cod *Gadus morhua *	1OKB	[151]

**Table 2:** Kinetic parameters for the hydrolysis of polysaccharides at 25°C by psychrophilic and mesophilic *α*-amylases. Adapted from [181].

Substrate	Psychrophilic *α*-amylase			Mesophilic *α*-amylase		
	*k*_cat_	*K*_*m*_	*k*_cat_/*K*_*m*_	*k*_cat_	*K*_*m*_	*k*_cat_/*K*_*m*_
	s^−1^	mg L^−1^	s^−1^ mg^−1^ L	s^−1^	mg L^−1^	s^−1^ mg^−1^ L
Starch	663	155	4.3	327	41	8.0
Amylopectin	636	258	2.5	222	53	4.2
Amylose	2148	178	12.1	700	36	19.4
Dextrin	716	586	1.2	311	61	5.1
Glycogen	491	1344	0.3	193	46	4.2

**Table 3:** Activation parameters of the hydrolytic reaction of *α*-amylases at 10°C. Adapted from [160].

	*k*_cat_	Δ*G* ^#^	Δ*H* ^#^	*T*Δ*S* ^#^
	s^−1^	kcal mol^−1^	kcal mol^−1^	kcal mol^−1^
Psychrophile	294	13.3	8.3	−5.0
Mesophile	97	14.0	11.1	−2.9
Thermophile	14	15.0	16.8	1.8

**Table 4:** Microcalorimetric parameters of thermal unfolding shown in Figure 9.

Proteins	*T*_*m*_ (°C)	Δ*H*_cal_ (kcal mol^−1^)
*α*-Amylases		
Psychrophilic	44	214
Mesophilic	66	319
Thermophilic	86	487
DNA-ligases		
Psychrophilic	33	46
Mesophilic	54	253
Thermophilic	95–101	413
